# The Impact of COVID-19 on Racial Disparities in Death Quality: The Moderating Role of Hospice and Palliative Care

**DOI:** 10.1093/geroni/igaf122.2318

**Published:** 2025-12-31

**Authors:** Clifford Ross, Brina Ratangee, Emily Schuler, Zheng (Noah) Lian, Benmun Damul, Deborah Carr, Lucie Kalousova

**Affiliations:** Vanderbilt University, Nashville, Tennessee, United States; Vanderbilt University, Nashville, Tennessee, United States; Vanderbilt University, Nashville, Tennessee, United States; Vanderbilt University, Nashville, Tennessee, United States; Vanderbilt University, Nashville, Tennessee, United States; Boston University, Boston, Massachusetts, United States; Vanderbilt University, Nashville, Tennessee, United States

## Abstract

**Objectives:**

This study investigates how the COVID-19 pandemic affected death quality and whether these impacts differed for White and Black decedents. We also evaluate the extent to which hospice and palliative care moderated these associations.

**Methods:**

Data are from the Health and Retirement Study (HRS) Core and Exit Interviews conducted between January 2018 and May 2021. Our analytic sample included 1,595 decedents (281 Black, 1,314 White). Outcomes include proxy-reported perceived death quality and whether decedents’ healthcare wishes were consistently met. Multivariable regression models with three-way interactions were used to evaluate the effects of race and hospice/palliative care usage on death quality during and before the COVID-19 pandemic.

**Results:**

Deaths during the pandemic were associated with lower subjective death quality scores and reduced odds of healthcare wishes being met. Proxies for Black decedents rated overall death quality higher than White proxies, although they also reported Black decedents were less likely to have their healthcare wishes fulfilled. Hospice care during the pandemic was linked to decreased death quality for Black decedents, likely reflecting disparities in the effectiveness of hospice/palliative care.

**Discussion:**

Death during (versus prior to) the COVID-19 pandemic was associated with worse death quality. The use of hospice/palliative care during COVID-19 was associated with poorer quality deaths for Blacks. Our results suggest that purportedly protective healthcare practices may be implemented less effectively for Black patients, underscoring the need for cultural competence training and sufficient structural resources for potentially underserved healthcare settings.

